# Effect of Salt Concentrations on the Proximate Composition, Microbial Load, and Sensory Attributes of Dry‐Salted *Labeobarbus* Species

**DOI:** 10.1002/fsn3.70867

**Published:** 2025-09-01

**Authors:** Solomon Birie, Minwyelet Mingist, Mulugeta Kibret, Tadlo Yitayew Atlog, Hirut Geremew, Banchiamlak Getnet

**Affiliations:** ^1^ Department of Fisheries and Aquatic Sciences, College of Agriculture and Environmental Sciences Bahir Dar University Bahir Dar Ethiopia; ^2^ Department of Biology, Faculty of Natural and Computational Sciences Debre Tabor University Debre Tabor Ethiopia; ^3^ Department of Biology, College of Science Bahir Dar University Bahir Dar Ethiopia; ^4^ Department of Food Engineering, Faculty of Chemical and Food Engineering, Bahir Dar Institute of Technology Bahir Dar University Bahir Dar Ethiopia; ^5^ Department of Animal Production & Technology, College of Agriculture and Environmental Science Bahir Dar University Bahir Dar Ethiopia

**Keywords:** dry salting, *Labeobarbus* spp., microbial, proximate, sensory

## Abstract

Salting is a traditional fish preservation method that reduces moisture content, enhances shelf life, and improves sensory attributes. In Lake Tana, inconsistent salt application by fishers affects preservation effectiveness, highlighting the need to determine optimal salt concentrations for improved fish quality. The objective of this study was to assess how varying salt concentrations influence the proximate composition, microbial counts, and sensory characteristics of *Labeobarbus* spp. Mature *Labeobarbus* spp. of similar length were purchased from the Bahir Dar landing site, then gutted, split into butterfly fillets, and washed. The fish were divided into three equal‐weight batches (~3 kg) and dry‐salted with coarsely ground commercial‐grade NaCl at concentrations of 10%, 20%, and 30% for 7 days. Then the dry‐salted products were analyzed for proximate composition, microbial load, and sensory attributes. The study found that higher salt concentrations in dry‐salted *Labeobarbus* spp. reduced moisture content (58.63% at 10% salt treatment to 45.42% at 30% salt treatment) with no significant difference between 20% and 30% salt treatments. The ash content increased, while fat and protein levels did not significantly differ between 20% and 30% treatments. Water activity decreased as salt concentration increased, likely enhancing preservation. Higher salt concentrations also reduced microbial load, with the 30% treatment showing the lowest bacterial load (3.06 log CFU/g). Sensory analysis indicated that the 10% salt treatment scored lower in most attributes, except color, compared to the 20% and 30% treatments, with scores ranging from 2.4 for odor at 10% to 7.6 for color at 20% and 30%. The experimental findings indicate that treatments with 20% and 30% salt concentrations result in desirable sensory attributes and acceptable microbial and nutritional quality.

## Graphical abstract

This study evaluated the effect of varying salt concentrations (10%, 20%, and 30%) on the quality of *Labeobarbus* spp. from Lake Tana. Higher salt levels reduced moisture content, water activity, and microbial load while improving ash content and sensory attributes. A 20% salt concentration was found to be optimal for achieving desirable product quality.

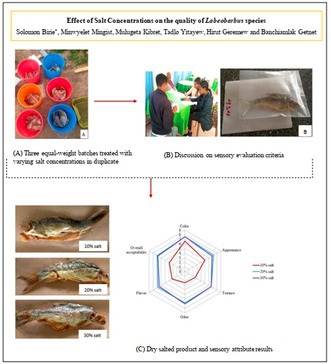

## 1 Introduction

Salting is an ancient fish preservation method that continues to be widely practiced in various regions around the world (Farid et al. 2016). In salted fish production, the raw fish is first headed, gutted, butterfly split, and washed, followed by salting using either pickle or kench methods (Horner 1992; Marchetti et al. 2021). In the kench method (dry salting), the brine that diffuses from the fish is allowed to drain off, but the brine is not drained off; rather, it immerses the fish in wet salting (pickling/brining) (Marchetti et al. 2021). The dry salting method works by lowering both moisture content and water activity, which extends shelf life and improves sensory attributes (Horner 1992; Andres et al. 2005; Farid et al. 2016; Emorine et al. 2021). Salting can be weak (salt content between 5% and 15%), medium (15%–25%), and strong (25%–30%) (Corrias et al. 2022). During salting, the salt concentration gradient is important for the movement of salt ions into the fish (Inguglia et al. 2018). Studies have shown that higher salt concentrations correlate with increased water loss in salted fish (Bras and Costa 2010). However, the diffusion of salt into the muscle and the quality of the final product depend on fat content, fillet thickness, fish size, temperature, salt concentration, salting method, and length of the salting period (Wang et al. 2000; Barat et al. 2006; Bellagha et al. 2007; Liang et al. 2021). Despite the development of alternative preservation techniques like refrigeration and freezing, fish salting remains a popular method due to its capacity to improve key sensory attributes, such as color, taste, flavor, and texture (Fan et al. 2014; Cropotova et al. 2021).

Lake Tana is home to 28 fish species, 18 of which are *Labeobarbus* spp., a group of highly valued fishery resources that support the livelihoods of thousands of fishermen (Anteneh et al. 2012; Gebremedhin et al. 2013). Salting remains a widely practiced traditional method of fish preservation in the lake during the rainy season of the year. However, fishers often apply table salt randomly without specific measurements. This rough estimate reduces the effectiveness of the preservation process, resulting in inconsistent quality and increased spoilage. Therefore, it is crucial to explore the impact of varying salt concentrations on fish quality to optimize salt levels for a higher‐quality product with desirable sensory attributes. Additionally, employing an appropriate salting method at its optimal level could extend shelf life, reduce fish loss, and create higher demand for the product. Despite this, limited data exist regarding the optimal salting conditions that ensure the best quality and sensory attributes for salted fish products in Lake Tana. Thus, this experimental study was designed to investigate the effects of salt concentration on the proximate composition, microbial load, and sensory attributes of dry‐salted *Labeobarbus* spp.

## 2 Materials and Methods

### 2.1 Chemicals and Reagents

All chemicals and reagents used in this study were of analytical grade. Sodium chloride (NaCl) was used for the dry‐salting of *Labeobarbus* spp. For protein content determination, concentrated sulfuric acid (H_2_SO_4_), Kjeldahl catalyst (CuSO_4_), 40% sodium hydroxide (NaOH), 4% boric acid (H_2_BO_3_), mixed indicators (bromocresol and methyl red), and hydrochloric acid (HCl) were used. Hexane was used for fat content analysis. Silver nitrate (AgNO_3_) and potassium chromate were used for salt determination. For microbial analysis, 0.1% sterile peptone water, 0.85% sterile saline solution, and Plate Count Agar (PCA) were used. Distilled water was used throughout for reagent preparation and dilution.

### 2.2 Sample Collection and Experimental Design

**FIGURE 1:**
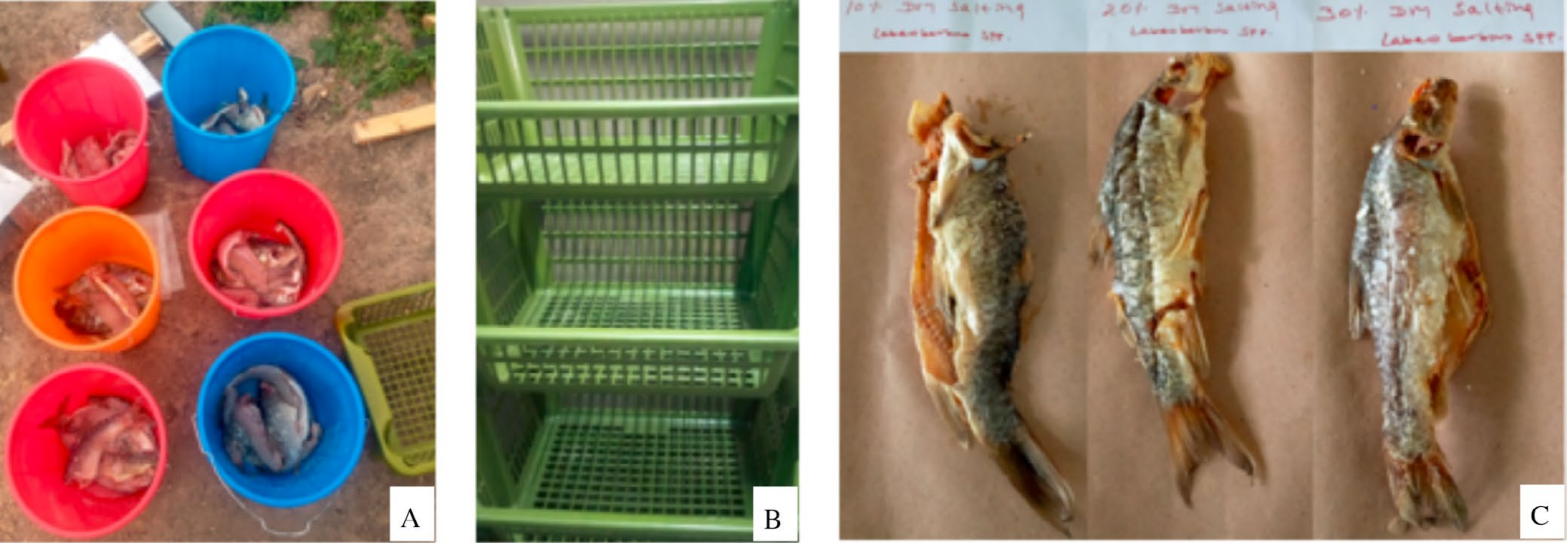
Dry salting process of *Labeobarbus* spp.: (A) Three equal‐weight batches treated with varying salt concentrations in duplicate, (B) perforated crates used to facilitate brine drainage during salting, and (C) the final dry‐salted fish products.

The study was carried out in 2023 at the Bahir Dar Institute of Technology (BiT), Bahir Dar University. For the dry salting experiment, mature and nearly equal‐sized individuals of *Labeobarbus* spp. were purchased from fishers at the Bahir Dar landing site. The length and weight of each fish were measured, and the fish were transported to the Food Processing Laboratory of BiT using an icebox. Upon arrival, the fish were thoroughly washed with clean water. The gills and internal organs were removed using knives, and the fish were split longitudinally into butterfly fillets. The fillets were washed again to remove residual blood and impurities. Before salting, a random subset of fillets was taken from the processed fish and set aside as the untreated group (control). These untreated samples were analyzed for proximate composition and microbial load to serve as a baseline for comparison with the salted treatments. The remaining fillets were divided into three equal batches (~3 kg each) and dry‐salted using salt concentrations equivalent to 10%, 20%, and 30% of the fish's weight (fish weight: salt weight). Each treatment was conducted in duplicate (Figure 1). Since the experimental units were mature, morphologically similar, and nearly uniform in size and weight, a Completely Randomized Design (CRD) was used to assign treatments. Coarsely ground, commercial‐grade sodium chloride was uniformly rubbed onto the surface and inner cavity of each fillet to ensure uniform salt absorption. Once salted, the fillets were arranged in single layers within perforated plastic crates to allow proper fluid drainage. To minimize dust and contamination during salting, the crates were covered with clean plastic sheets and kept under ambient environmental conditions for 7 days. After this dry‐salting period, the products were subjected to proximate, microbial, and sensory analyses.

### 2.3 Proximate Analysis

From each batch, three individuals of dry‐salted *Labeobarbus* spp. fillet were randomly selected, and the muscle tissue was sliced, minced, pooled, and homogenized. This sample was analyzed in triplicate for proximate composition (moisture, fat, protein, and ash) following the specific instructions outlined in the Association of Official Analytical Chemists (AOAC 2000) manual.

#### 2.3.1 Moisture Content

Following the AOAC (2000) manual, the moisture content was calculated as:

$$\text{Moisture}\;\left(\%\right)=\frac{W1-W2}{W1}\times 100$$

where, W1 = weight (g) of sample before drying; W2 = weight (g) of sample after drying.

#### 2.3.2 Ash Content

As per the AOAC (2000) manual, the ash content was calculated as:

$$\mathrm{Ash}\;\left(\%\right)=\frac{\text{Weight of}\;\mathrm{ash}}{\text{Weight of sample}}\times 100$$

#### 2.3.3 Protein Content

The crude protein was determined using the Kjeldahl method, as outlined by AOAC (2000). The total nitrogen and crude protein content of the sample was calculated using the formulas:

$$\%N=\frac{\left(\text{Volume of}\;\mathrm{HCl}\;\text{for sample titration}-\text{Volume of}\;\mathrm{HCl}\;\text{used for blank titration}\right)\times M\times 14.01}{\text{Weight of sample}\;}\times 100$$

$$\text{Crude protein}\;\left(\%\right)=\%N\times 6.25$$

where, %*N* is nitrogen content in percent; M is the normality of HCl; 14.01 is the molecular weight of nitrogen; 6.25 is a conversion factor.

#### 2.3.4 Fat Content

The fat content was determined using the Soxhlet method, following AOAC (2000) guidelines, and calculated as:

$$\text{Crude}\;\mathrm{fat}\;\left(\%\right)=\frac{\text{Weight of extracted}\;\mathrm{fat}}{\text{Weight of sample}}\times 100$$

### 2.4 Physicochemical Analysis

#### 2.4.1 Determination of Salt Concentration

The salt concentration was measured by applying the Mohr titration technique. The percentage of NaCl was calculated using the formula:

$$\text{NaCl}\;\left(\%\right)=\frac{\text{Titer value}\times \text{Normality of AgNO}3\times 58.4\times 100}{\text{Weight of the sample}\times 1000}$$

#### 2.4.2 Determination of Water Activity

Water activity of the fish samples was determined using an AQUALAB 4TE water activity meter (USA, Serial Number: S40003072).

### 2.5 Determination of the Microbial Load

For microbial analysis, muscle tissue from three randomly selected fillets of each batch was sliced and minced using a sterile knife, then pooled and homogenized. Then, 25 g of the homogenized tissue was measured under aseptic conditions inside the biosafety hood and mixed with 225 mL of 0.1% sterile peptone and normal saline solution (0.85% NaCl) in a sterilized plastic bag. The mixture was homogenized for 2 min using a Stomacher 400 circulator (England, Serial No. 42110). Subsequently, tenfold serial dilutions ranging from 10^−2^ to 10^−7^ were prepared using sterile peptone and saline solutions with a sterile pipette. The aerobic mesophilic bacteria were obtained by pour‐plating 1 mL of the homogenized sample onto sterile plate count agar (HiMedia, India), which was then incubated at 37°C for 24–48 h. Plates with 30–300 colony‐forming units were counted to determine the total load of aerobic mesophilic bacteria (Maturin and Peeler 2001). The recorded result was expressed as the mean colony‐forming units (CFU) per gram using the following formula (Maturin and Peeler 2001):

$$N=\frac{\sum C}{\left[\left(1\times n1\right)+\left(0.1\times n2\right)\right]d}$$

where *N* = number of colonies per gram; ∑C = sum of counted colonies; n1 = number of plates counted in the first dilution; n2 = number of plates counted in the second dilution; *d* = dilution of the first count obtained.

### 2.6 Sensory Analysis

**FIGURE 2:**
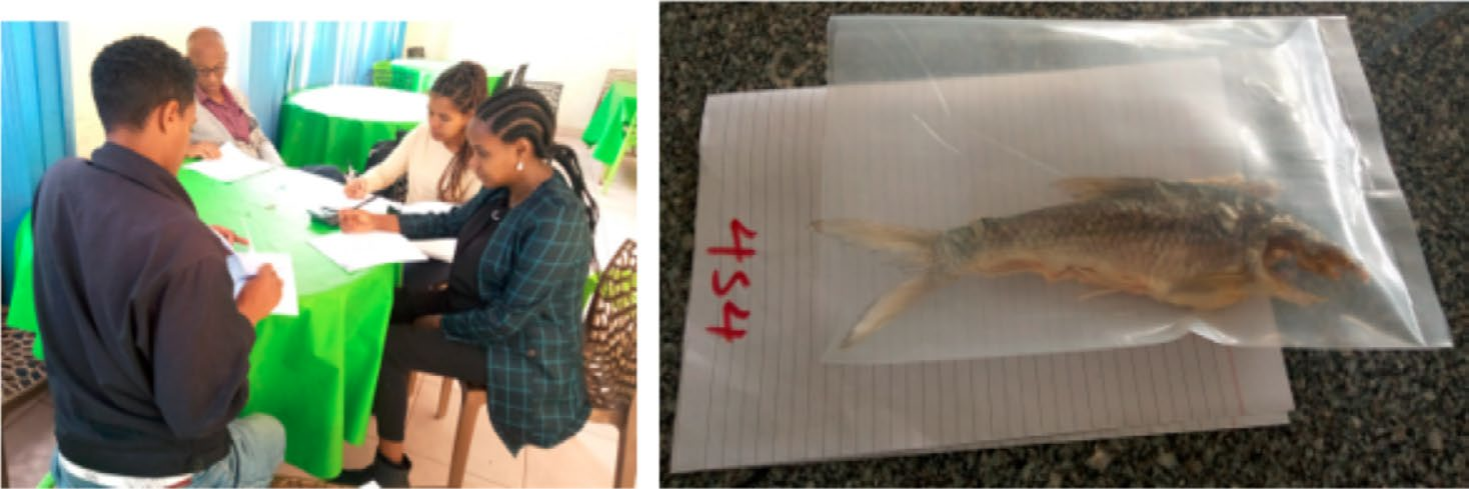
Team members' discussion on sensory evaluation criteria for dry‐salted *Labeobarbus* spp. products.

For sensory evaluation, three individual dry‐salted *Labeobarbus* spp. from each batch were randomly selected and independently assessed. A panel of six evaluators from the staff at Bahir Dar University assessed sensory attributes such as color, appearance, flavor, texture, and odor. Each attribute was determined by every panelist using a nine‐point preference hedonic scale. Samples were placed at room temperature on a clean surface in odorless plastic containers labeled with randomly assigned three‐digit codes (Figure 2). Panelists rated the samples on a 9‐point hedonic scale, ranging from 1 (dislike extremely) to 9 (like extremely) (Lawless and Heymann 2010). The sensory evaluation was conducted in accordance with the ethical and professional guidelines outlined by the Institute of Food Science and Technology (IFST 2020) for sensory analysis of food products. For flavor assessment, a slice cut from the muscle tissues of the sample was delivered to the assessors, and water was used to rinse the mouth between samples as mentioned by Heir et al. (2022). Scores for each sensory attribute were combined, and the mean value was reported as the product's overall acceptability score. A score of 9–7 points indicates very good quality, a score of 6.9–5 points indicates good or acceptable quality, and a score of 4.9–1 points indicates unacceptable quality (Meilgaard et al. 1999; Lawless and Heymann 2010).

### 2.7 Statistical Analysis

Statistical analyses were performed using SPSS software (Version 26.0). Descriptive statistics including means, percentages, and standard deviations were calculated. Data visualization was done through graphs and tables. Microbial counts were log‐transformed and expressed as log_10_ (CFU/g). Differences in proximate composition, microbial load, and sensory attributes among salt concentration treatments were evaluated using one‐way ANOVA. Statistical significance was considered at *p* < 0.05.

## 3 Results and Discussion

### 3.1 Effect of Salt Concentration on Proximate Composition

Dry‐salted *Labeobarbus* spp. with 10%, 20%, and 30% salt resulted in moisture contents of 58.63% ± 0.71%, 48.20% ± 1.12%, and 45.42% ± 1.44%, respectively (Table 1). This demonstrates a decreasing trend in moisture content with increasing salt concentration. Similar findings have been reported by Farid et al. (2016) and Khalifah et al. (2022). For example, Farid et al. (2016) observed a 28.19% moisture reduction after 7 days of dry salting in the shoal. The moisture reduction observed in this study might be associated with osmotic dehydration, where salt draws water from the fish muscle, resulting in a reduced moisture content (Bras and Costa 2010). However, the difference in moisture content between the 20% and 30% salt treatments was not statistically significant (*p* > 0.05). The higher moisture content observed in the 10% salt treatment may increase the risk of microbial growth and spoilage, as the associated water activity remains relatively high (Table 1). In contrast, the 20% and 30% salt treatments reduced moisture levels to below 50%, emphasizing the importance of applying adequate salt concentrations during dry salting to enhance product safety. According to Kumar et al. (2017), salting and drying can enhance the rate of water loss, improving the physicochemical stability of fish products. These results are consistent with previous studies by Ariyarathna and Porarinsdottir (2011), Ibrahim (2014), and Jeyasanta et al. (2016), which demonstrated that salting significantly reduces moisture content and thereby supports the preservation of fish products. Overall, the study suggests that salt concentrations of at least 20% are necessary to achieve effective moisture reduction and ensure microbial safety in dry‐salted *Labeobarbus* spp. products.

**TABLE 1:** Proximate composition and physicochemical properties of dry‐salted *Labeobarbus* spp. treated under different concentrations of sodium chloride.

Treatments	Moisture (%)	Ash (%)	Fat (%)	Protein (%)	Water activity	Salt content (%)
10% NaCl	58.63 ± 0.71^b^	15.18 ± 0.23^b^	7.95 ± 0.59^b^	16.32 ± 0.23^b^	0.85 ± 0.01^b^	8.22 ± 0.36^b^
20% NaCl	48.20 ± 1.12^a^	20.60 ± 0.42^c^	8.5 ± 0.20^bc^	21.31 ± 0.39^c^	0.74 ± 0.05^a^	15.45 ± 0.09^c^
30% NaCl	45.42 ± 0.44^a^	22.63 ± 0.37^d^	8.97 ± 0.27^c^	21.59 ± 0.22^c^	0.74 ± 0.01^a^	18.45 ± 0.62^d^
Untreated	80.75 ± 0.72^c^	1.28 ± 0.03^a^	4.93 ± 0.82^a^	12.60 ± 0.60^a^	0.99 ± 0.01^c^	0.32 ± 0.025^a^

*Note:* The data were expressed as Mean ± SD (*n* = 3); Lowercase superscript (a–d) indicates statistical difference among the mean values of the salt treatments in the same column using a one‐way ANOVA (*p* < 0.05).

The ash content of dry‐salted *Labeobarbus* spp. products treated with 10%, 20%, and 30% salt concentrations was 15.18% ± 0.23%, 20.60% ± 0.42%, and 22.63% ± 0.37%, respectively, while the ash content of the untreated product was only 1.28% ± 0.03%. These significantly higher ash values in salted samples may be associated with the higher salt absorption and the water loss that occurs during the salting process (Fitri et al. 2022). This finding is in agreement with findings reported by Talab and Ghanem (2021) and Farid et al. (2016), who observed similar trends in various salted fish species. However, the ash values recorded in the present study are lower than those reported by Khalifah et al. (2022), possibly due to species differences and salting duration. Similarly, the higher ash content in dry‐salted fish has also been reported by Farid et al. (2016) and Kumar et al. (2017). The results from this study suggest that increasing salt levels during dry salting significantly enhance the mineral content of dry‐salted *Labeobarbus* spp., thereby improving both preservation potential and product quality. However, this study did not include detailed mineral profiling. Future research is recommended to use spectroscopic techniques to quantify specific mineral elements. This would allow for a more precise assessment of mineral variation across different salting levels and provide critical insights into the functional roles and potential health implications of salted fish products.

The fat content of dry‐salted *Labeobarbus* spp. products treated with 10%, 20%, and 30% salt concentrations showed a significant increase compared to the untreated samples (Table 1). This increase in fat content is likely due to the reduction in moisture caused by salting, which concentrates the remaining components. The fat content at 20% salting was 8.5% ± 0.20%, which is higher than the value reported by Khalifah et al. (2022) under a similar salting level. Additionally, Farid et al. (2016) also reported lower fat content (3.99%) at a 30% salt concentration. These variations may be attributed to species‐specific differences, environmental factors, or pre‐harvest conditions such as diet, maturity, or seasonal variation (Shirai et al. 2002; Chakraborty et al. 2015). It is also important to note that fat content in salted fish can be affected not only by salting concentration but also by post‐salting storage conditions. As reported by Zhao et al. (2020), prolonged storage or high salt exposure can trigger lipolysis, leading to partial breakdown and loss of crude fat. Thus, balancing salt concentration and optimizing storage time are critical not only for maintaining nutritional quality but also for preserving the sensory and safety attributes of the final product.

The protein content of dry‐salted *Labeobarbus* spp. products treated with 10%, 20%, and 30% salt concentrations showed a significant increase compared to the untreated samples, which had a protein content of 12.60% ± 0.60% (Table 1). This increase in protein content is likely associated with the dehydration effect of salting, where the removal of water reduces the overall weight of the fish, thereby concentrating the remaining nutrients (Kim et al. 2020). However, no statistically significant differences in protein content were observed between the 20% and 30% salt treatments. The protein content recorded at 20% salt concentration was higher than that reported by Khalifah et al. (2022), but lower than the value reported by Farid et al. (2016) for fish treated with 30% salt. Contrasting reports in the literature have indicated that salting may lead to protein degradation or denaturation. For example, Kumar et al. (2017) reported that salt‐treated fish had lower protein levels than unsalted fish, potentially due to protein denaturation during extended drying or storage. In this study, however, dry salting was carried out for only 7 days, which likely minimized protein degradation. Supporting this, Farid et al. (2014) observed that significant protein loss typically occurs after prolonged storage. Future studies should investigate the long‐term effects of salting and storage on protein integrity of dry‐salted *Labeobarbus* spp.

### 3.2 Effect of Salt Concentration on Physicochemical Properties

The mean salt content of the dry‐salted *Labeobarbus* spp. products treated with 10%, 20%, and 30% salting concentrations was 8.22% ± 0.36%, 15.45% ± 0.09%, and 18.45% ± 0.62%, respectively (Table 1). These values are higher than those reported by Birie et al. (2025) for traditionally salted, sun‐dried products around Lake Tana. However, the salt content observed at the 20% treatment level aligns with the findings reported by Khalifah et al. (2022). The observed increase in salt content with increasing salt levels indicates effective diffusion of sodium chloride into the fish muscle. This diffusion is crucial not only for flavor development but also for reducing moisture content and lowering water activity. From a nutritional standpoint, increased salt content reflects greater dietary salt levels, which may improve product preservation and flavor but could pose concerns for sodium‐sensitive individuals. Therefore, it is essential to balance salt concentration not only to enhance microbial stability and shelf life but also to ensure the nutritional suitability of the final product.

The water activity of dry‐salted *Labeobarbus* spp. products declined significantly compared to the untreated samples (Table 1). At 20% salting, the water activity was 0.74 ± 0.05. Although this is slightly higher than the value reported by Khalifah et al. (2022), it remains within a safe range for inhibiting the growth of most spoilage bacteria. The observed differences in water activity may be associated with variations in salt‐to‐fish contact time, which influences the efficiency of salt diffusion and moisture removal (Corrias et al. 2022). Microbiologically, water activity plays a critical role in determining the shelf stability of dried fish. Most bacteria require a minimum water activity value of 0.85 to grow (Fontana 2020), and the values observed in both 20% and 30% salt treatments are well below this. However, it is important to note that certain microorganisms such as molds and yeasts can survive and proliferate at lower water activity values (Fontana 2020). Therefore, while the reduction in water activity achieved through dry salting offers substantial preservation benefits, continuous monitoring and proper storage are essential to prevent spoilage by more tolerant microorganisms.

### 3.3 Effect of Salt Concentration on Microbial Load

The aerobic mesophilic bacterial load in dry‐salted *Labeobarbus* spp. products treated with 10%, 20%, and 30% salt concentrations was 5.12 ± 0.05, 5.03 ± 0.01, and 3.06 ± 0.04 log CFU/g, respectively. All of these values were lower than the load found in the untreated sample, which was 6.15 log CFU/g. These findings indicate a clear inverse relationship between salt concentration and microbial load, indicating the antimicrobial effect of higher salt levels. This trend aligns with the observations of Hong et al. (2012), who reported a decrease in spoilage microorganisms in salted fish. Comparable microbial counts have been reported by Sivaraman and Sivam (2015), who found 4.81 log CFU/g in salted dried fish, while Khalifah et al. (2022) reported slightly higher microbial loads at 20% salting levels, possibly due to differences in species, environmental conditions, or handling practices. In contrast, Turan et al. (2006) reported a lower load (2.66 log CFU/g) in salted fish products stored for 1 month. The marked reduction in microbial load at 30% salting highlights its effectiveness in creating an unfavorable environment for bacterial growth, primarily due to the significant reduction in water activity and moisture content. Although the 10% salt treatment showed higher bacterial counts, the values remained within acceptable limits defined by the International Commission on Microbiological Specifications for Foods (ICMSF 1986).

### 3.4 Effect of Salt Concentration on Sensory Attributes

The sensory scores for all attributes of 10% salt‐treated *Labeobarbus* spp. products were significantly lower than those for the 20% and 30% treatments, except color (*p* < 0.05). The scores for the attributes analyzed ranged from a minimum of 2.4% for the odor attribute in the 10% treatment to a maximum of 7.6% for the color attribute in both the 20% and 30% treatments (Table 2). These findings suggest that increasing the salt concentration positively influenced the sensory characteristics of the fish, while lower salt levels were associated with diminished sensory attributes, particularly in odor.

**TABLE 2:** Sensory analysis of dry‐salted *Labeobarbus* spp. treated under different concentrations of sodium chloride.

Treatments	Sensory attributes					
	Color	Appearance	Texture	Odor	Flavor	Acceptance
10% NaCl	6.6 ± 0.89^a^	5.0 ± 0.99^a^	2.6 ± 0.89^a^	2.4 ± 1.67^a^	3.2 ± 1.64^a^	3.9 ± 0.99^a^
20% NaCl	7.6 ± 1.14^a^	7.2 ± 1.09^b^	6.6 ± 1.51^b^	6.8 ± 1.09^b^	6.8 ± 0.83^b^	7.0 ± 1.02^b^
30% NaCl	7.6 ± 1.14^a^	6.8 ± 1.64^b^	6.6 ± 2.07^b^	7.0 ± 1.87^b^	6.4 ± 2.51^b^	6.8 ± 1.81^b^

*Note:* The data were expressed as Mean ± SD (*n* = 6); Lowercase superscript (a, b) indicates statistical difference among the mean values of the salt treatments in the same column using a one‐way ANOVA (*p* < 0.05).

**FIGURE 3:**
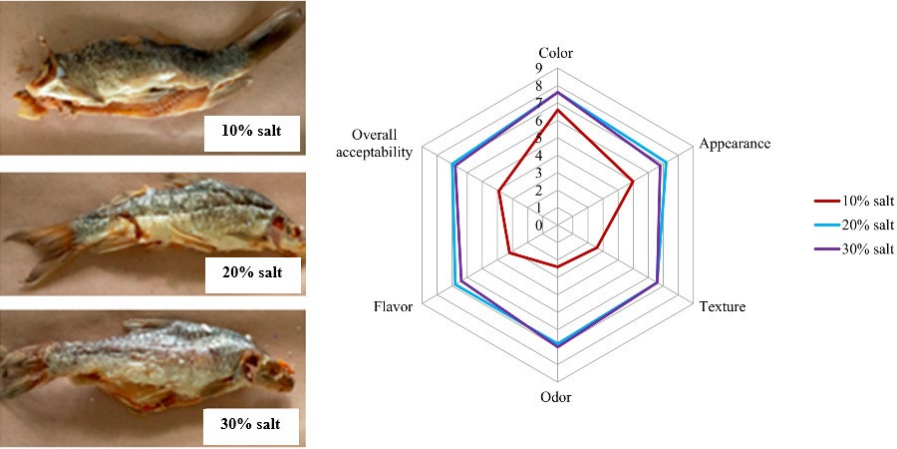
Image of dry‐salted *Labeobarbus* spp. products (left side) and its radar chart for sensory evaluation (right side) at 10%, 20%, and 30% salt concentrations.

The dry‐salted *Labeobarbus* spp. products exhibited a whitish to yellowish color (Figure 3), with sensory scores of 6.6, 7.6, and 7.6 for the 10%, 20%, and 30% salt treatments, respectively. Whitish, yellowish, and light brown colors are reported to be indicators of good‐quality dried fish products (Huque et al. 2013; Mithun et al. 2021). The color change during salting might be associated with a reduction in water content (Stien et al. 2005; Bras and Costa 2010).

The *Labeobarbus* spp. treated with a 30% salt concentration received the highest average rating for odor, although this score was not significantly different from that of the 20% salt‐treated products (*p* > 0.05) (Table 2). For texture, the fish treated with 20% and 30% salt concentrations received the highest score, while the 10% salt‐treated fish exhibited a softer texture, with an average score of 2.6. These findings agree with Barat et al. (2003), who observed that salting improves fish firmness, with the effect being weaker at lower salt levels. The sensory score for flavor was lower for the 10% salt treatment but higher for the 20% treatment, which was liked by panelists. Based on overall appearance, fish treated with 10% salt look less attractive compared to those treated with 20% and 30% salt concentrations. This reduced attractiveness might be associated with compromised odor and texture. The findings of this assessment revealed that the sensory attributes of fish treated with 20% and 30% salt concentrations were nearly similar but significantly differed from the 10% treatment, as shown in Figure 3.

## 4 Conclusions

This experimental study revealed that higher salt concentration treatment in *Labeobarbus* spp. effectively enhances preservation by reducing moisture content, water activity, and microbial load while also improving mineral composition. The 30% salt treatment, in particular, created an environment less favorable for bacterial growth, contributing to both better shelf life and quality. Additionally, higher salt concentrations (20% and 30%) consistently improved sensory attributes, particularly odor and texture, while the 10% treatment scored lower in overall sensory quality, although color remained consistent across all treatments. The findings suggest that although dry‐salted *Labeobarbus* spp. treated with 20% and 30% salt concentrations provide desirable sensory attributes and acceptable microbial and nutritional quality, the 20% treatment is considered more appropriate in light of public health concerns regarding high salt intake, as it balances product quality with consumer health and satisfaction. Further research is recommended to assess the long‐term storage stability of dry‐salted fish treated with these salt concentrations.

## Author Contributions

**Solomon Birie:** conceptualization (equal), data curation (equal), formal analysis (equal), investigation (equal), methodology (equal), writing – review and editing (equal). **Minwyelet Mingist:** conceptualization (equal), supervision (equal), writing – review and editing (equal). **Mulugeta Kibret:** conceptualization (equal), supervision (equal), writing – review and editing (equal). **Tadlo Yitayew Atlog:** investigation (equal), methodology (equal), writing – review and editing (equal). **Hirut Geremew:** methodology (equal), writing – review and editing (equal). **Banchiamlak Getnet:** methodology (equal), writing – review and editing (equal).

## Ethics Statement

This study was approved by the Food and Nutrition Research Center of Bahir Dar University. No approval number was issued. Fish samples were obtained from catches made by local fishers, and no live specimens were handled or sacrificed. The research complied with all relevant ethical standards for the use of animal‐derived samples in scientific studies.

## Conflicts of Interest

The authors declare no conflicts of interest.

## Data Availability

The datasets generated and analyzed during this study are available from the corresponding author upon reasonable request.
